# Supplementary material for: Could the mitotic count improve personalized prognosis in melanoma patients?
Source: PLoS One. 2024 Apr 16;19(4):e0302309. doi: 10.1371/journal.pone.0302309 (PMC11020877; doi:10.1371/journal.pone.0302309)
Supplement: Attachment — Submitted filename: 2023 12 07 Response to Reviewers.docx [file pone.0302309.s001.docx]

**Journal requirements:**

When submitting your revision, we need you to address these additional requirements.

1. Please ensure that your manuscript meets PLOS ONE's style requirements, including those for file naming. The PLOS ONE style templates can be found at

<https://journals.plos.org/plosone/s/file?id=wjVg/PLOSOne_formatting_sample_main_body.pdf> and

<https://journals.plos.org/plosone/s/file?id=ba62/PLOSOne_formatting_sample_title_authors_affiliations.pdf>

***Authors: Done.***

2. Our staff editors have determined that your manuscript is likely within the scope of our Personalised Medicine Call for Papers. This call for papers aims to showcase the breadth of research within personalized medicine and its impact on innovating the healthcare horizon. Additional information can be found on our announcement page: Personalized Medicine Call for Paper- PLOS Collection

If you would like your manuscript to be considered for this collection, please let us know in your cover letter and we will ensure that your paper is treated as if you were responding to this call. Please note that being considered for the Collection does not require additional peer review beyond the journal’s standard process and will not delay the publication of your manuscript if it is accepted by PLOS ONE. If you would prefer to remove your manuscript from collection consideration, please specify this in the cover letter.

***Authors: We would be honoured to have our article considered in the collection Personalized Medicine, please see our latest cover letter.***

3. We note that the grant information you provided in the ‘Funding Information’ and ‘Financial Disclosure’ sections do not match.

When you resubmit, please ensure that you provide the correct grant numbers for the awards you received for your study in the ‘Funding Information’ section.

***Authors: Done.***

4. We note that you have indicated that data from this study are available upon request. PLOS only allows data to be available upon request if there are legal or ethical restrictions on sharing data publicly. For more information on unacceptable data access restrictions, please see <http://journals.plos.org/plosone/s/data-availability#loc-unacceptable-data-access-restrictions>.

In your revised cover letter, please address the following prompts:

a) If there are ethical or legal restrictions on sharing a de-identified data set, please explain them in detail (e.g., data contain potentially sensitive information, data are owned by a third-party organization, etc.) and who has imposed them (e.g., an ethics committee). Please also provide contact information for a data access committee, ethics committee, or other institutional body to which data requests may be sent.

b) If there are no restrictions, please upload the minimal anonymized data set necessary to replicate your study findings as either Supporting Information files or to a stable, public repository and provide us with the relevant URLs, DOIs, or accession numbers. For a list of acceptable repositories, please see <http://journals.plos.org/plosone/s/data-availability#loc-recommended-repositories>.

We will update your Data Availability statement on your behalf to reflect the information you provide.

***Authors: We made available the minimal anonymized data set necessary to replicate our findings at the link https://doi.org/10.6084/m9.figshare.24637938.v1 .***

**Additional Editor Comments** :

The introduction has to be re-­‐structured, and the rationale for the study should be set clear. The manuscript should also be checked for types and grammars errors.

***Authors: Thank you for the suggestions. We have carefully reviewed the entire manuscript and had the text corrected by our language and editing service.***

**Comments to the Author**

1. Is the manuscript technically sound, and do the data support the conclusions?

The manuscript must describe a technically sound piece of scientific research with data that supports the conclusions. Experiments must have been conducted rigorously, with appropriate controls, replication, and sample sizes. The conclusions must be drawn appropriately based on the data presented.

Reviewer #1: Yes

Reviewer #2: Yes

***Authors: Thank you.***

2. Has the statistical analysis been performed appropriately and rigorously?

Reviewer #1: Yes

Reviewer #2: Yes

***Authors: Thank you.***

3. Have the authors made all data underlying the findings in their manuscript fully available?

The [PLOS Data policy](http://www.plosone.org/static/policies.action#sharing) requires authors to make all data underlying the findings described in their manuscript fully available without restriction, with rare exception (please refer to the Data Availability Statement in the manuscript PDF file). The data should be provided as part of the manuscript or its supporting information, or deposited to a public repository. For example, in addition to summary statistics, the data points behind means, medians and variance measures should be available. If there are restrictions on publicly sharing data—e.g. participant privacy or use of data from a third party—those must be specified.

Reviewer #1: Yes

Reviewer #2: Yes

***Authors: Thank you.***

4. Is the manuscript presented in an intelligible fashion and written in standard English?

PLOS ONE does not copyedit accepted manuscripts, so the language in submitted articles must be clear, correct, and unambiguous. Any typographical or grammatical errors should be corrected at revision, so please note any specific errors here.

Reviewer #1: Yes

Reviewer #2: Yes

***Authors: Thank you.***

5. Review Comments to the Author

Please use the space provided to explain your answers to the questions above. You may also include additional comments for the author, including concerns about dual publication, research ethics, or publication ethics. (Please upload your review as an attachment if it exceeds 20,000 characters)

***Reviewer #1***: This is an interesting study looking at the prognostic role of Mitoses in melanoma patients. This topic has been discussed in the past years , since it gave indication to SLN biopsy in the last AJCC classification (upstaging the T1a to T1b). The study is well conducted and the results and discussion sound interesting. There authors made correction with SLNB status but not with the involvement of SLN (micro macro metastasis). Please have a look at Quaglino et al. Surg Oncol. 2011 Dec;20(4):259-64. Is there a particular reason not to look at this association? Please discuss.

***Authors: Thank you for the positive feedback. We agree on the importance that SLN involvement could potentially have at the prognostic level. Unfortunately, micro/macro metastasis classification is not available in our registries. We have added this aspect in the study limitation paragraph properly citing Quaglino et al.***

***Reviewer #2***: In this manuscript the authors examine the prognostic significance of mitotic rate in two cohorts of melanoma patients from the Veneto Cancer Registry. They show a correlation between increasing mitotic rate and various histopathologic factors in melanoma. They also show worse survival with increasing mitotic rate by Kaplan-Meier analysis. They investigate various cutoffs for mitotic rate in the entire cohorts, and in individual T categories. They conclude that mitotic rate does have prognostic significance and may be used in individualized prognostic assessments of melanoma patients. Overall, the manuscript is useful in its reinforcement of the importance of mitotic rate. Attention to the following points is warranted to strengthen their conclusions, and also to address limitations in the available cohorts that precluded additional observations regarding the prognostic significance of mitotic rate.

1. A critical issue regarding assessment of mitotic rate, given the non-linear shape of its impact on survival, is the identification of optimal cut-points, both in the entire cohort of patients, as well as in specific T categories, as has been previously demonstrated (ref 14). To begin with, this should be discussed in greater detail in the Discussion section. While the authors examine this issue in some analyses, additional analyses are suggested. For example, in their K-M analysis of the entire cohort in Fig. 1, the authors examine different cutpoints for mitotic rate (0, 1, 2, 3-5; and >6). This analysis suggests the following “optimal” grouping or index for mitotic rate with differing survival: 0-2; 3-5; and > 6. It would be both interesting and important to analyze this further, both in univariate and multivariate analyses for the entire cohort. Also, given that the analysis of individual cutpoints only showed significant results in T2 patients, it would be interesting to examine this index in each T category to determine whether it would show a significant prognostic significance.

***Authors: Thanks for the interesting insight into the nonlinear form of MR impact on survival and challenges in optimal cut-points identification. We have added a paragraph regarding this in the discussion. In addition, we did further regression analysis to estimate the effects of mitotic count on survival using the “optimal” grouping 0-2; 3-5; and ≥6 (Table 3). The results, both using group encoding, both using single cut-offs for MR, are now reported for the entire sample and for each value of T, from T1 to T4 (Tables 3 and 4).***

2. For the multivariate analysis of the entire cohort, it would be important to show the significance of mitotic rate (using the best individual cutpoint or the index mentioned in point 1 above) along with the other factors analyzed, so that its impact can be understood in the context of the other factors available.

***Authors: Thank you for this important advice. Age and TNM stage were found to remain strong predictors of survival, but also mitotic rate, together with site, histology, and sex turned out to be statistically associated with prognosis. In Table 3 we have now added the HR estimates and p values for the adjustment variables.***

3. As indicated above, there are significant limitations to the cohorts available that may preclude additional significant observations regarding the prognostic significance of mitotic rate in this analysis. One limitation is the preponderance of stage I cases (76% of the cohort), which in part accounts for 50% of the cases having a mitotic index of 0. Therefore, the proportion of cases with elevated mitotic rate is low. In addition, this results in smaller sample sizes for T3 and T4 cases, precluding a meaningful assessment of the prognostic impact in these subsets. These issues should be explicitly discussed.

***Authors: Thank you for pointing out this issue. We added the following sentence in the study limitation paragraph to address this aspect:***

*[…] being a population study, a further limitation of the present work is the underrepresentation of subjects with T3 or T4 melanoma at diagnosis. Low sample size may have hindered statistical significance and power in these subpopulations. In our region (Veneto, Italy), prevention campaigns over the past 30 years have increased population awareness about pigmented lesions and, together with early diagnosis, have indeed significantly reduced the incidence of advanced-stage melanomas. On the other hand, we believe that the analysis of a big group of T1 and T2 patients, such as our, allowed interesting observations on MR value, particularly for T2 stage […] could lead to the proposal of a MR based personalized planning […] for T2N0 patients (IB and IIA stage), including the highly debated adjuvant therapy [16].*

4. What is the median follow up of the two cohorts? This should be explicitly stated, as it represents another major limitation of the study, especially in view of the predominance of stage I lesions, which can present with late relapses and deaths (i.e., beyond five years).

***Authors: Thank you for the suggestion. In Table 1, we included the follow-up years statistics, and we added the following sentence in the results section:***

*The overall median follow-up time was 3.82 years (min-max 0.01 - 6.00). Stratifyng by T stage it was 3.95, 3.75, 3.63, and 3.11 years respectively for T1, T2, T3, and T4 subjects.*

5. Table 1- 46% of patients were male. This is in contrast to most studies, which show a male predominance. Do the authors have any explanation for this finding?

**Authors: The ASCO Cancer Net Editorial Board (03/2023) document on melanoma states that “The average age at diagnosis is 65. [...] Before age 50, more women are diagnosed with melanoma than men”. Concordantly, in a recent population-based study on Victorian Cancer Registry data (Australia), the authors report that before 50 years old, melanoma diagnosis is more prevalent in females (51.8%) over males (48.2%) (https://www.cancervic.org.au/research/vcr/cancer-fact-sheets/melanoma.html#how-common-is-melanoma). Similar results are also shown in the latest SEER update of delay-adjusted CMM incidence rate trends (**[**https://seer.cancer.gov/statistics-network/explorer/application.html?site=53&data_type=1&graph_type=2&compareBy=sex&chk_sex_3=3&chk_sex_2=2&rate_type=2&race=1&age_range=9&stage=101&advopt_precision=1&advopt_show_ci=on&hdn_view=0&advopt_show_apc=on&advopt_display=2#resultsRegion0**](https://seer.cancer.gov/statistics-network/explorer/application.html?site=53&data_type=1&graph_type=2&compareBy=sex&chk_sex_3=3&chk_sex_2=2&rate_type=2&race=1&age_range=9&stage=101&advopt_precision=1&advopt_show_ci=on&hdn_view=0&advopt_show_apc=on&advopt_display=2#resultsRegion0)**). The median age of our sample is 59 years (much less than 65, as above reported) and this might be an explanation for this finding.**

6. PLOS authors have the option to publish the peer review history of their article ([what does this mean?](https://journals.plos.org/plosone/s/editorial-and-peer-review-process#loc-peer-review-history)). If published, this will include your full peer review and any attached files.

If you choose “no”, your identity will remain anonymous but your review may still be made public.

**Do you want your identity to be public for this peer review?** For information about this choice, including consent withdrawal, please see our [Privacy Policy](https://www.plos.org/privacy-policy).

Reviewer #1: No

Reviewer #2: No

[NOTE: If reviewer comments were submitted as an attachment file, they will be attached to this email and accessible via the submission site. Please log into your account, locate the manuscript record, and check for the action link "View Attachments". If this link does not appear, there are no attachment files.]

While revising your submission, please upload your figure files to the Preflight Analysis and Conversion Engine (PACE) digital diagnostic tool, <https://pacev2.apexcovantage.com/>. PACE helps ensure that figures meet PLOS requirements. To use PACE, you must first register as a user. Registration is free. Then, login and navigate to the UPLOAD tab, where you will find detailed instructions on how to use the tool. If you encounter any issues or have any questions when using PACE, please email PLOS at [figures@plos.org](mailto:figures@plos.org). Please note that Supporting Information files do not need this step.
