# Supplementary material for: Could the mitotic count improve personalized prognosis in melanoma patients?
Source: PLoS One. 2024 Apr 16;19(4):e0302309. doi: 10.1371/journal.pone.0302309 (PMC11020877; doi:10.1371/journal.pone.0302309)
Supplement: Attachment — Submitted filename: 2024 03 12 Response to Reviewers.docx [file pone.0302309.s002.docx]

**Authors’ response to Review Comments to the Author**

**Reviewer #3:** The assessed manuscript was a 'Revised Manuscript with Track Changes' in the document PONE-D-22-26758_R1_reviewer. In general, the objective is clear, and the methodology is appropriate.

- In the abstract, the authors should add the time of overall survival evaluated (3-year overall survival) and use the term 'histological subtype' instead of 'type of morphology'.

**Authors: Ok, done. Thank you.**

- In Statistical Methods, make it clear that the considered period for short-term overall survival was 3 years.

**Authors: Ok, done. Thank you.**

- In Tables 1 and 2, it is important to include in the caption what 'Malignant (NOS)' means, once it is not a histological subtype of melanoma.

**Authors: Ok, abbreviations meaning are now specified in all the tables. Thank you.**

- In the title of Table 2, there is no need for the phrase “p-values < 0.05 are reported in bold” **Authors: Ok, deleted. Thank you.**
- In Table 2, the percentage is not consistently represented (Some values have two numbers after the decimal point, others do not).

**Authors: Zeros were originally omitted, resulting in inconsistent representation (number of digits). Now all estimates are reported with 2 digits after decimal point. Thank you.**

- Fig.1 is mentioned in the text, the title appears, but I could not find either the figure or its caption.

**Authors: Images were not included in the main manuscript as per PLOS ONE guidelines. Only figure captions were originally shown. Fig 1 and caption are now clearly reported in the Results section.**
Tables 3 and 4: highlight significant p-values in the adjusted model using bold, not red color.

**Authors: Ok, done. Thank you.**

- In the discussion, it is more appropriate to write "increased mitoses per mm2 are associated with a higher risk of mortality, particularly in T2 patients," instead of "mitoses per mm2 are associated with an increased risk of mortality, particularly in T2 patients."

**Authors: Amended as suggested, thank you.**

- In the discussion, it is more appropriate to write "In particular, we observe that patients with a high mitotic rate are more likely to be older, male, have a melanoma lesion with a vertical growth pattern, nodular histological subtype, ulceration, greater tumor thickness, and an advanced stage," instead of "In particular, we observe that patients with a high mitotic rate are more likely to be older, male, have a melanoma lesion with a vertical growth pattern, ulceration, greater tumor thickness, and an advanced stage."

**Authors: Amended as suggested, thank you.**

- In the discussion, "... the presence of pre-existing nevi were found to be typical of lesions with sparse mitotic activity," however, there is no information about the absence or presence of pre-existing nevi in the results. Please, insert the data or remove this sentence.
  **Authors: We rephrased the sentence. Thank you for the correction.**
- In the discussion, reference 14 appears in the wrong place (page 72 of the document PONE-D-22-26758_R1_reviewer).

**Authors: We thoroughly checked references citation numbers. Thank you for pointing out this error.**

- In the last paragraph of the discussion, it should be T2N0M0, not T2N0.

**Authors: Corrected. Thank you.**
